# Comparing Geant4 physics models for proton-induced dose deposition and radiolysis enhancement from a gold nanoparticle

**DOI:** 10.1038/s41598-022-05748-0

**Published:** 2022-02-02

**Authors:** Saeed Rajabpour, Hassan Saberi, Javad Rasouli, Nasrollah Jabbari

**Affiliations:** 1 Department of Medical Physics, Faculty of Medicine, Urmia University of Medical Sciences, Urmia, Iran; 2 Solid Tumor Research Center, Cellular and Molecular Medicine Institue, Urmia University of Medical Sciences, Urmia, Iran; 3 Solid Tumor Research Center, Cellular and Molecular Medicine Institute, Urmia University of Medical Sciences, Urmia, Iran

**Keywords:** Biological techniques, Biophysics, Cancer, Medical research, Oncology, Nanoscience and technology, Physics

## Abstract

Gold nanoparticles (GNPs) are materials that make the tumor cells more radiosensitive when irradiated with ionizing radiation. The present study aimed to evaluate the impact of different physical interaction models on the dose calculations and radiochemical results around the GNP. By applying the Geant4 Monte Carlo (MC) toolkit, a single 50-nm GNP was simulated, which was immersed in a water phantom and irradiated with 5, 50, and 150 MeV proton beams. The present work assessed various parameters including the secondary electron spectra, secondary photon spectra, radial dose distribution (RDD), dose enhancement factor (DEF), and radiochemical yields around the GNP. The results with an acceptable statistical uncertainty of less than 1% indicated that low-energy electrons deriving from the ionization process formed a significant part of the total number of secondary particles generated in the presence of GNP; the Penelope model produced a larger number of these electrons by a factor of about 30%. Discrepancies of the secondary electron spectrum between Livermore and Penelope were more obvious at energies of less than 1 keV and reached the factor of about 30% at energies between 250 eV and 1 keV. The RDDs for Livermore and Penelope models were very similar with small variations within the first 6 nm from NP surface by a factor of 10%. In addition, neither the G-value nor the REF was affected by the choice of physical interaction models with the same energy cut-off. This work illustrated the similarity of the Livermore and Penelope models (within 15%) available in Geant4 for future simulation studies of GNP enhanced proton therapy with physical, physicochemical, and chemical mechanisms.

## Introduction

Radiotherapy is a common modality for the treatment of malignant diseases. The ultimate aim of photon radiotherapy is to provide energy deposition within a tumor using secondary electrons while concomitantly sparing normal tissues and the organs at risk. One major limitation of photon-based radiation therapy is the lacking selectivity of dose deposition in tumor tissues^1,2^.

Proton radiotherapy is a high-dose conformity radiation therapy technique that provides desirable and homogeneous dose coverage of the tumor volume within spread-out Bragg peak (SOBP) allowing for sparing of sensitive normal tissues with similar tumor control outcomes. The width and depth of the SOBP region have a direct relationship with the beam energy and the medium heterogeneity in the path of the proton beam. This region is generally considered to have the same relative biological effectiveness (RBE)/linear energy transfer (LET), except the more complicated dose fall-off region^3,4^. The advantage of proton therapy over photon and electron therapy is the high therapeutic ratio as a result of the high tumor control probability (TCP) and low normal tissue complication probability (NTCP)^4–7^.

Nanoparticle (NP)-aided radiation therapy has been proposed as an innovative approach in cancer treatment. Gold nanoparticles (GNPs) are materials with high atomic numbers (Z) produced with lengths ranging from 1 to 100 nm, which are designed to make the cancer cells more radiosensitive when irradiated with ionizing radiation^8,9^. GNPs are selectively delivered to the tumor sites through the mechanism of the enhanced permeability and retention effect (EPR)^10^. Most of the solid tumors show a higher amount of vascular permeability factor than normal tissues because of the large gaps between endothelial cells in tumor blood vessels^11^. External proton beams interact with these GNPs and, subsequently, lead to low-energy electron emissions, fluorescent photons, as well as generation of reactive species, which can increase the ionization within a small volume around the GNP and induce DNA damage and tumor cell killing^12–14^. Experimental studies have shown the improvement of the radiation therapy outcomes with protons and GNPs^12,15^.

Many studies have been performed based on the Monte Carlo (MC) simulations to quantify the direct and indirect DNA damages around NPs in combination with radiation^13,16,17^. Monte Carlo simulations have the potential to study the microdosimetry and radiochemical effects of ionizing radiation at the DNA scale. Geant4 is MC toolkit^18,19^ that offers a set of low-energy electromagnetic physics models (Livermore and Penelope) for simulating the photon and electron transport in different materials, including metallic nanoparticles down to sub-keV energies^20^. To estimate the direct physical damage and indirect chemical damage induced by ionizing radiation in the biological medium as liquid water, Geant4-DNA extension has been developed^21–24^.

Simulation of particle tracks and radiolysis of liquid water in Geant4-DNA are divided into different stages that follow each other in time: the physical stage (< 10^−15^ s), in which all the physical interactions such as ionization or excitation by primary or secondary particles, take place; the physicochemical stage (10^−15^–10^−12^ s), in which chemical bonds break and create new chemical species; and the chemical stage (10^−12^–10^−6^ s), in which the new chemical species diffuse, interact with each other, or react with DNA molecules, and lead to indirect DNA damage^16,23^.

Comparison of physics models for NP-aided radiotherapy simulation studies provides valuable insight into the medical physics community for NP-aided radiotherapy studies since they benefit from Geant4-DNA physics models for simulating the particle transport within water and Livermore or Penelope physics models for the NP area (mixed physics). The “Livermore” and “Penelope” “Physics List” include so-called condensed history (CH) algorithms to calculate the energy loss of charged particles. In this fashion, the cumulative effect of a number of interactions is used, instead of the interactions of charged particle in an event-by-event method^20^.

Previous simulation studies have revealed the different results of physical dose and direct damage as a result of the different physical interaction models^20,25,26^. These comparative studies only consider the microscale physics parameters of radiobiological effects; however, modeling the differences of direct damage alone cannot explain experimental proton therapy results.

The methodology of the current study is similar to the one adopted in the previous study by Tran et al.^13^, in which the Livermore physics list was investigated. However, the present work compares the Penelope and Livermore physics lists with each other. In addition, there are no published simulation data which could state the differences of radiochemical yields deriving from secondary electron production when Livermore and Penelope models are chosen for GNP.

Therefore, this study aimed to evaluate the impact of different physical interaction models of Geant4 (Penelope and Livermore) on the direct and indirect proton irradiation effects. In this work, parameters such as the abundance of electrons and photons, secondary electrons and photons spectra, radial dose distribution (RDD), dose enhancement factor (DEF), time-dependent G-values of radiolytic species, and radiolysis enhancement factor (REF) under proton irradiation were simulated with different energies (5, 50, and 150 MeV) using Geant4.

## Results

### Simulation validation

**Figure 1:**
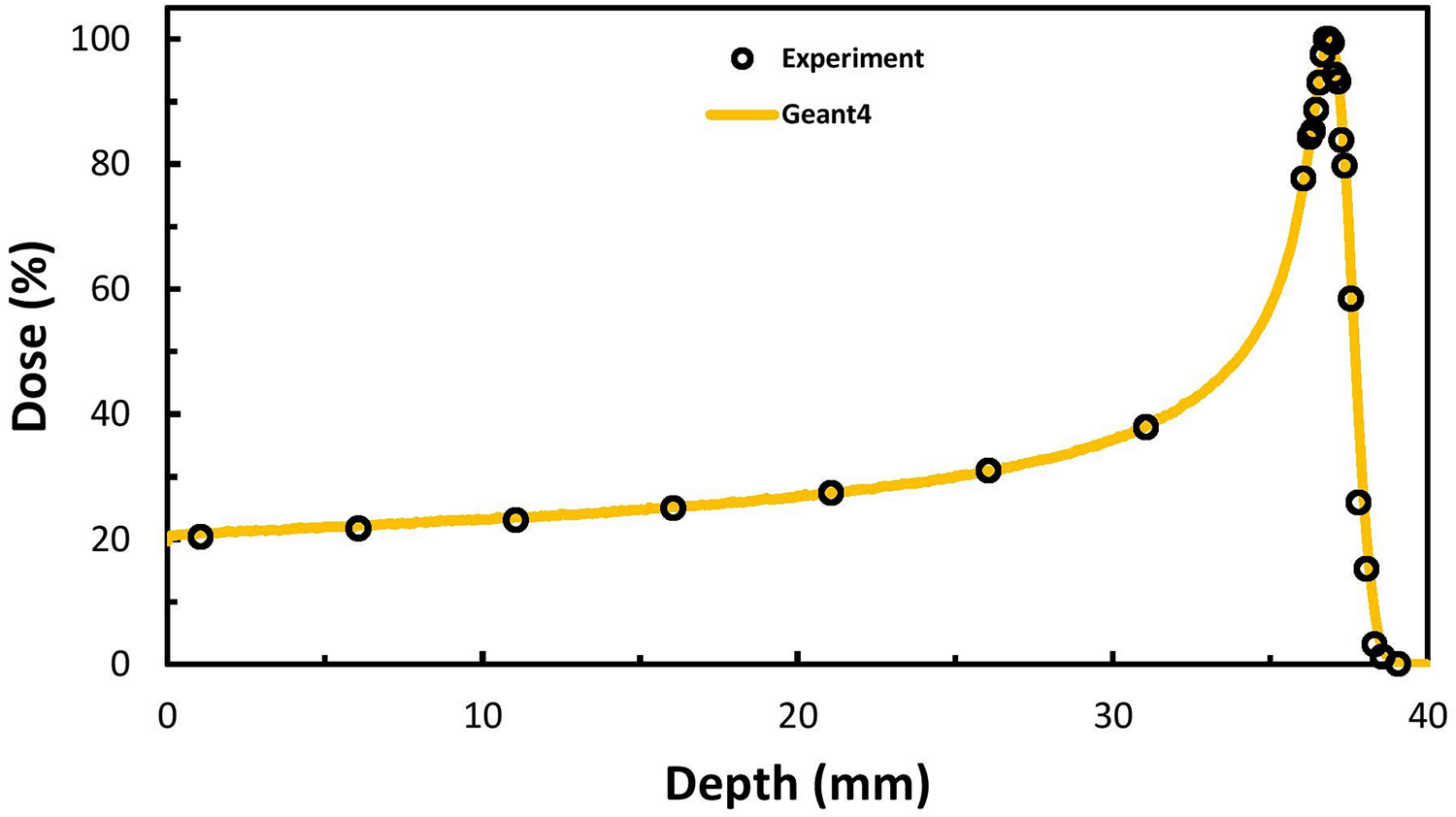
Simulation validation: Geant4 and experimental data from Faddegon et al.^27^ for the 67.5-MeV protons Bragg curve.

In the physical stage, the simulation results of the proton Bragg curve in water were compared with the experimental data from a previous study^27^ (Fig. 1). Considering uncertainties in MC calculations (less than 1%), the resulting maximum of less than 1% along the curve was acceptable for our Geant4 simulation model.

**Figure 2:**
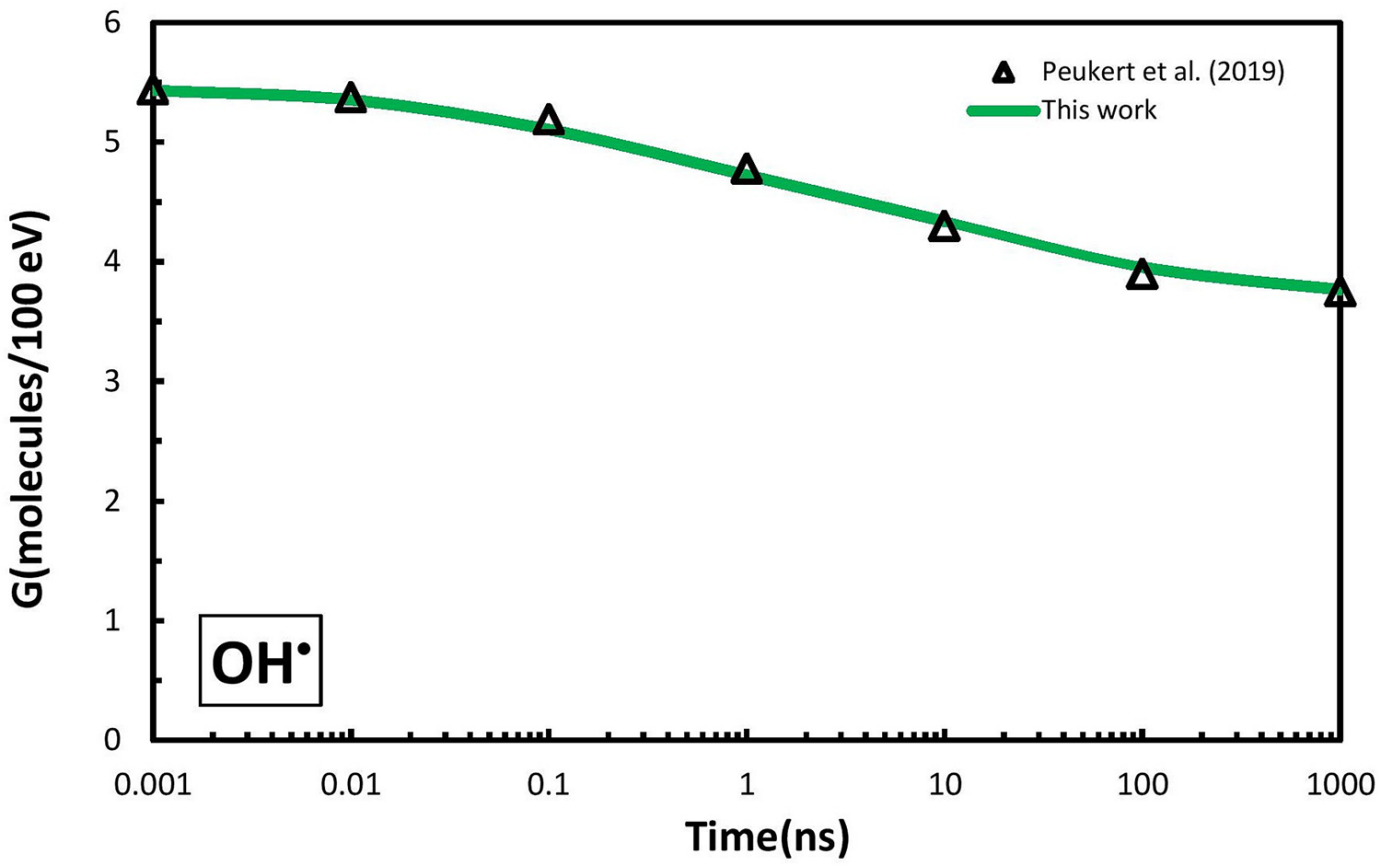
Simulation validation: comparison between the results of our simulation and the simulation by Peukert et al.^14^ for time-dependent G-value of hydroxyl radical around the GNP for 50-MeV proton beam.

In the chemical stage, the simulation results of the G-value of the hydroxyl radical were compared with the Geant4 data from a previous study^14^. These radicals were chosen for validation because these products of water radiolysis had the most severe reaction with DNA molecules. As shown in Fig. 2, there was good agreement between the Geant4 simulation results with less than 1% differences at each time point.

### Number of secondary particles

The total number of secondary particles emitting from the GNP following irradiation by 5, 50, and 150 MeV incident protons are shown in Table 1, along with the calculated differences between the selected models. For the comparison, we applied the same cut-off energy of 100 eV for the selected models as an internal threshold in the models. The impact of protons on GNP produced the secondary particles including Auger electrons, electrons deriving from the ionization process, characteristic photons and bremsstrahlung X-rays. Low energy electrons deriving from the ionization process formed a significant part of the total number of secondary particles generated in the presence of GNP. The Penelope model showed a larger number of these electrons, but lower Auger electrons and photons than the Livermore model. The discrepancies between the Livermore and Penelope models for all the particle types were dependent on the primary proton energies. The total number of secondary particles decreased as the incident proton kinetic energy increased with the exception of characteristic photons. In addition, characteristic photons showed a larger yield than Auger electrons for all the incident protons.

**Table 1:** The total number of secondary particles (statistical uncertainty < 1%) around GNP based on Livermore and Penelope models obtained for 5, 50, and 150-MeV proton beams.

Energy (MeV)	Particle/proton				
	Model	Ionization electrons*	Auger electrons	Characteristic X-Ray	Bremsstrahlung X-Ray
5	Livermore	0.712	4.04 × 10^−5^	1.34 × 10^−5^	4.53 × 10^−5^
	Penelope	1.030	3.6 × 10^−5^	1.15 × 10^−5^	3.92 × 10^−5^
	%Dev.^a^	30.874	12.222	16.522	15.561
50	Livermore	0.080	4.83 × 10^−6^	3.6 × 10^−5^	4.98 × 10^−6^
	Penelope	0.115	4.7 × 10^−6^	3.55 × 10^−5^	4.45 × 10^−6^
	%Dev.^a^	30.435	2.766	1.408	11.911
150	Livermore	0.031	2.2 × 10^−6^	1.91 × 10^−5^	1.9 × 10^−6^
	Penelope	0.044	2.2 × 10^−6^	1.89 × 10^−5^	1.9 × 10^−6^
	%Dev.^a^	29.545	< 0. 001	1.058	< 0. 001

^a^Deviation between Livermore and Penelope.

*Electrons deriving from the ionization process.

### Secondary electron spectra

**Figure 3:**
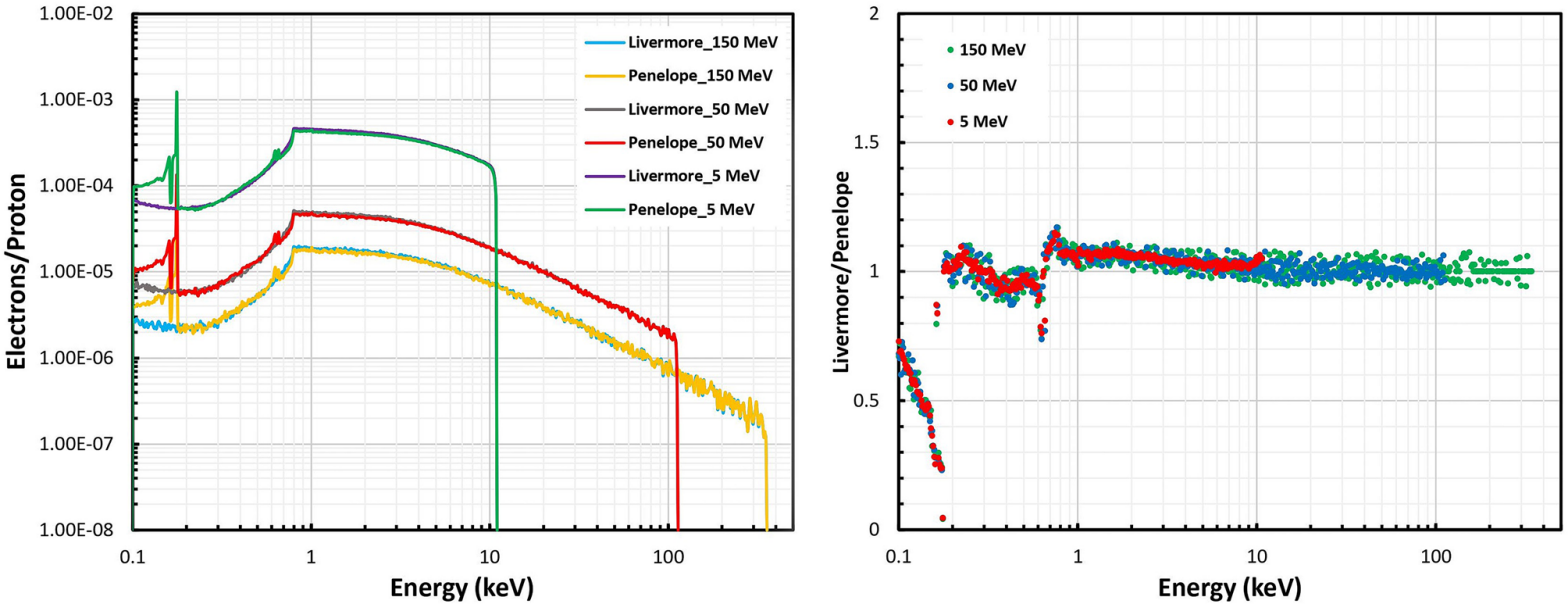
Comparison of the secondary electron spectra around the GNP between the Livermore and Penelope models obtained for 5, 50, and 150-MeV proton beams.

The impact of the physical interaction models (Livermore and Penelope) on the secondary electron spectra emitting from the GNP was calculated, as shown in Fig. 3, for the 5, 50, and 150 MeV initial proton energies as well as the ratios of the selected models. Discrepancies of the secondary electron spectrum between Livermore and Penelope were more obvious at the energies less than 1 keV and reached the factor of about 30% at the energies between 250 eV and 1 keV. Within these energy ranges, the Penelope model produced a larger number of low-energy secondary electrons than the Livermore model. In the spectra using the Penelope models the largest value occurred just less than 0.2 keV; however for the spectra using the Livermore model and outside the peak in the Penelope case, the spectra from ~ 1 to ~ 10 keV resulted in more electrons/protons than those less than 1 keV.

The differences between the Livermore and Penelope models were not affected by the primary proton energies. The maximum energy transferred to secondary electrons by 5, 50, and 150 MeV protons was about 11, 112, and 352 keV, respectively. For all the initial proton energies, the number of secondary electrons increased up to the energy of 0.8 keV, then decreased slightly up to the maximum energy of secondary electrons.

### Secondary photon spectra

**Figure 4:**
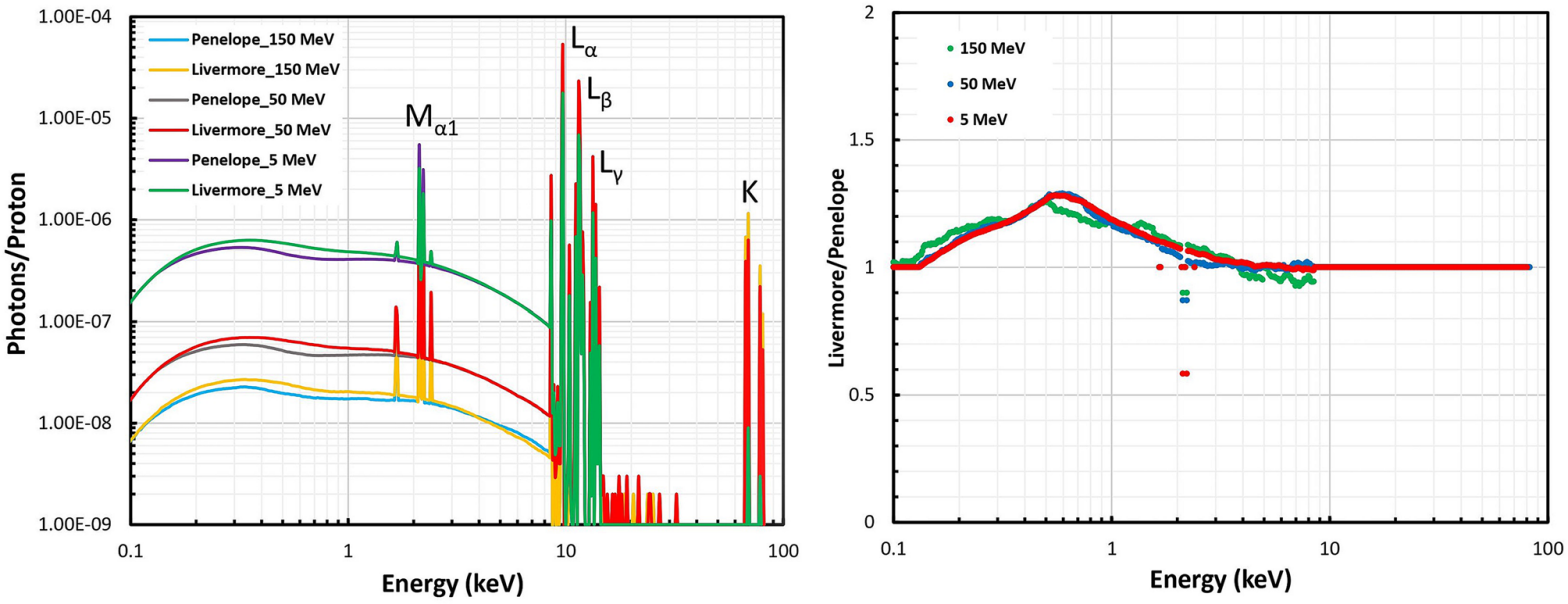
Comparison of the secondary photon spectra around the GNP between the Livermore and Penelope models obtained for 5, 50, and 150-MeV proton beams.

The impact of the physical interaction models (Livermore and Penelope) on the secondary photons emitting from the GNP was calculated as shown in Fig. 4 for the 5, 50, and 150 MeV initial proton energies as well as the ratios of the selected models. All the incident proton energies showed M_α1_ (2.1 keV), L (from 9.7 to 13.4 keV), and K (from 68 to 78 keV) de-excitation lines as well as bremsstrahlung background. The L de-excitation lines were dominant characteristic photons for all the incident proton energies. The physical interaction model selection only affected the M_α1_ emission line by a factor of about 40%, 13%, and 10% for 5, 50, and 150 MeV proton energies, respectively. These differences were observed for peak height, while the peak position and width were similar for the selected physical interaction models. The differences of the bremsstrahlung emission based on two models reached the factor of 30% around 0.5 keV for all the initial proton energies. High energy protons produced lower photon yield.

### Radial dose distribution

**Figure 5:**
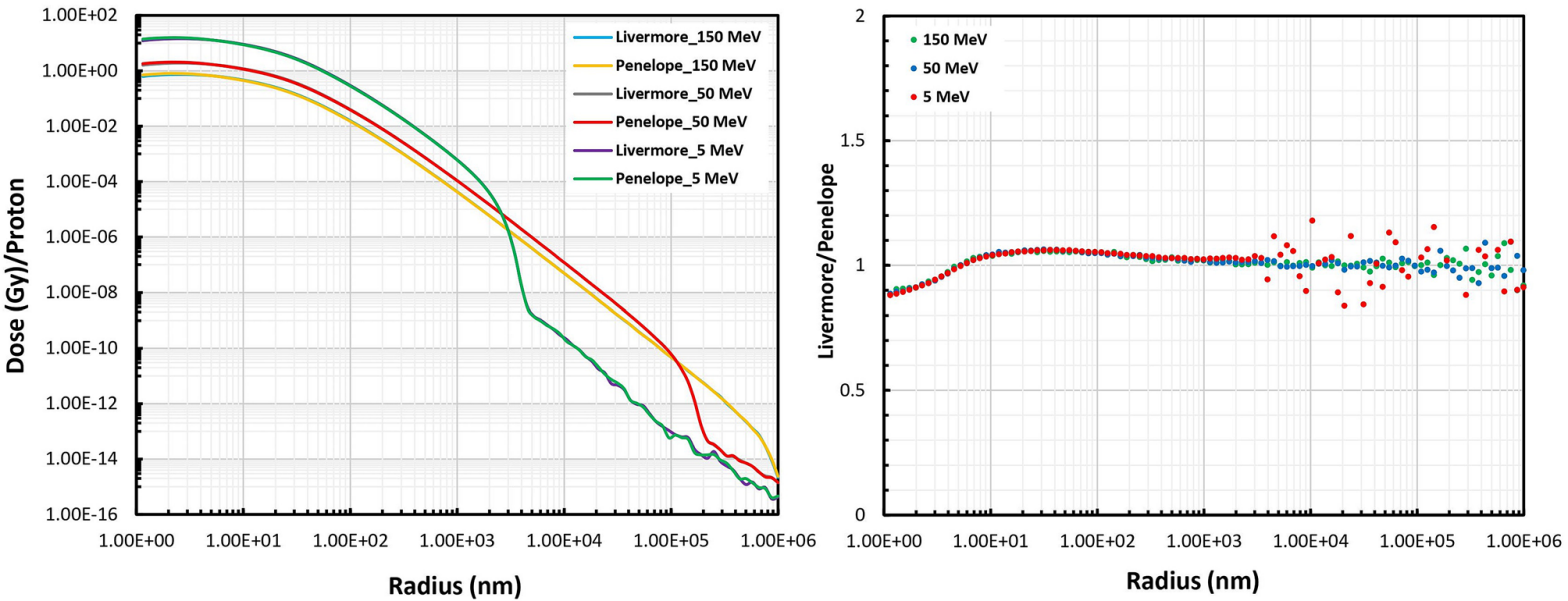
Comparison of the RDD between the Livermore and Penelope models obtained for 5, 50, and 150-MeV proton beams as a function of radial distance from the GNP surface.

The impact of the physical interaction models on the RDD as a function of radial distance from the GNP surface, which was irradiated with 5, 50, and 150 MeV proton energies and scaled to the number of incident protons is shown in Fig. 5, along with the ratios of the selected models. In all the cases, the RDD quickly decreased as a function of radial distance. The RDDs for Livermore and Penelope models were very similar and there was no appreciable difference between the selections of these models for all the three proton energies. It should be noted that the RDD for Penelope was slightly higher than that for Livermore within the first 6 nm up to a factor of 10%. Past approximately 6 nm the RDD for Livermore was slightly higher up to a factor of 6%. With regard to proton energies, the discrepancies were independent of initial proton energy. In addition, those secondary particles released from GNP upon 5 MeV proton irradiation had the highest deposited dose from GNP surface up to about 3 µm, while the secondary particles originating from 50 and 150 MeV irradiation led to the highest deposited dose in the range of 3–100 µm and 100–1000 µm, respectively.

### Dose enhancement factor

**Figure 6:**
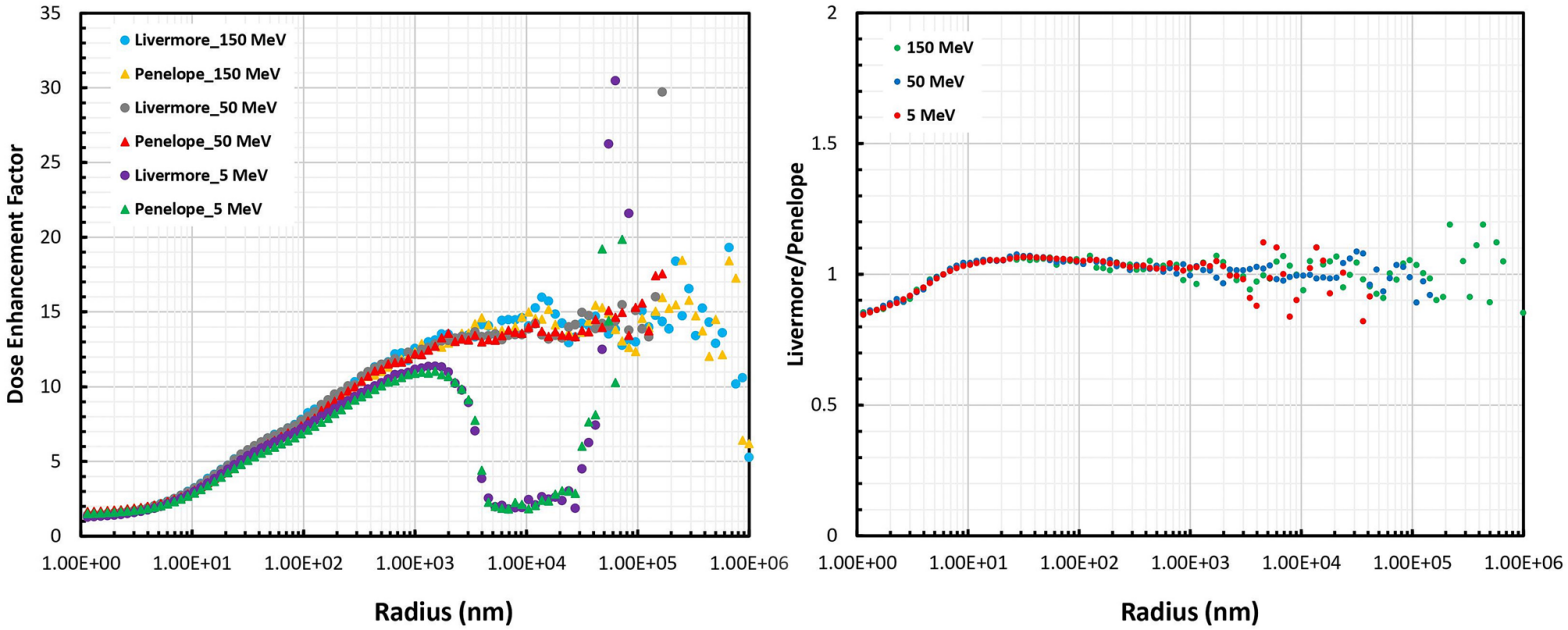
Comparison of the DEF between the Livermore and Penelope models obtained for 5, 50, and 150-MeV proton beams as a function of radial distance from the GNP surface.

Figure 6 presents the comparison of the DEF between Livermore and Penelope obtained for 5, 50, and 150 MeV proton beams as a function of radial distance from the GNP surface as well as the ratios of the two models. In the range of 6 nm, the Penelope model showed higher DEF by the factor up to about 15% and after 6 nm, the DEF for Livermore was slightly higher up to a factor of 7%. As presented in the right panel of Fig. 6, the differences were independent of the initial proton energy. In all the cases, the DEF values were more than 1, which indicated that the absorbed dose induced by the presence of the GNP increased compared with irradiation without the GNP. The 5 MeV proton irradiation had the DEF increasing from 1.4 to 11 at the distance of about 1 µm, then decreased sharply and increased again when approaching 30 µm. However, the 50 and 150 MeV proton irradiations had the DEF increasing from about 1.5 to 14 until the maximum ranges of the secondary particles.

### Time-dependent G-values

**Figure 7:**
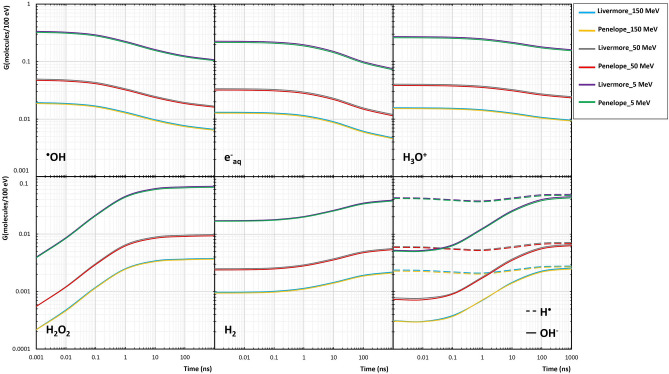
Comparison of the G-value (molecules/100 eV) between the Livermore and Penelope models obtained for 5, 50, 150-MeV proton beams as a function of time.

Figure 7 illustrates the influence of selecting physical interactions (Livermore and Penelope) on the chemical stage by comparing the time-dependent G-values of the most important reactive species of water radiolysis, including e_aq_^−^, ^·^OH, H^·^, H_3_O^+^, H_2_, OH^−^, and H_2_O_2_, in the proximity of the GNP irradiated with 5, 50, and 150 MeV incident protons. The G-values were calculated in the time intervals of 1 ps to 1 µs after the initial energy deposition. The G-values of all the radiochemical species were not affected by selecting physical interaction models. In addition, there was an inverse relationship between the G-values and the incident proton energy. The chart trend of H_3_O^+^, e_aq_^−^, and ^·^OH species, which were produced at the pre-chemical stage (before 1 ps), decreased with the increase of time, except for H^·^ with a slight variation. In addition, the chart trend of H_2_, OH^−^, and H_2_O_2_, which were created at the chemical stage (1 ps to 1 µs) as a product of the reactions between the reactive species, increased with the time increase.

### Radiolysis enhancement factor

**Figure 8:**
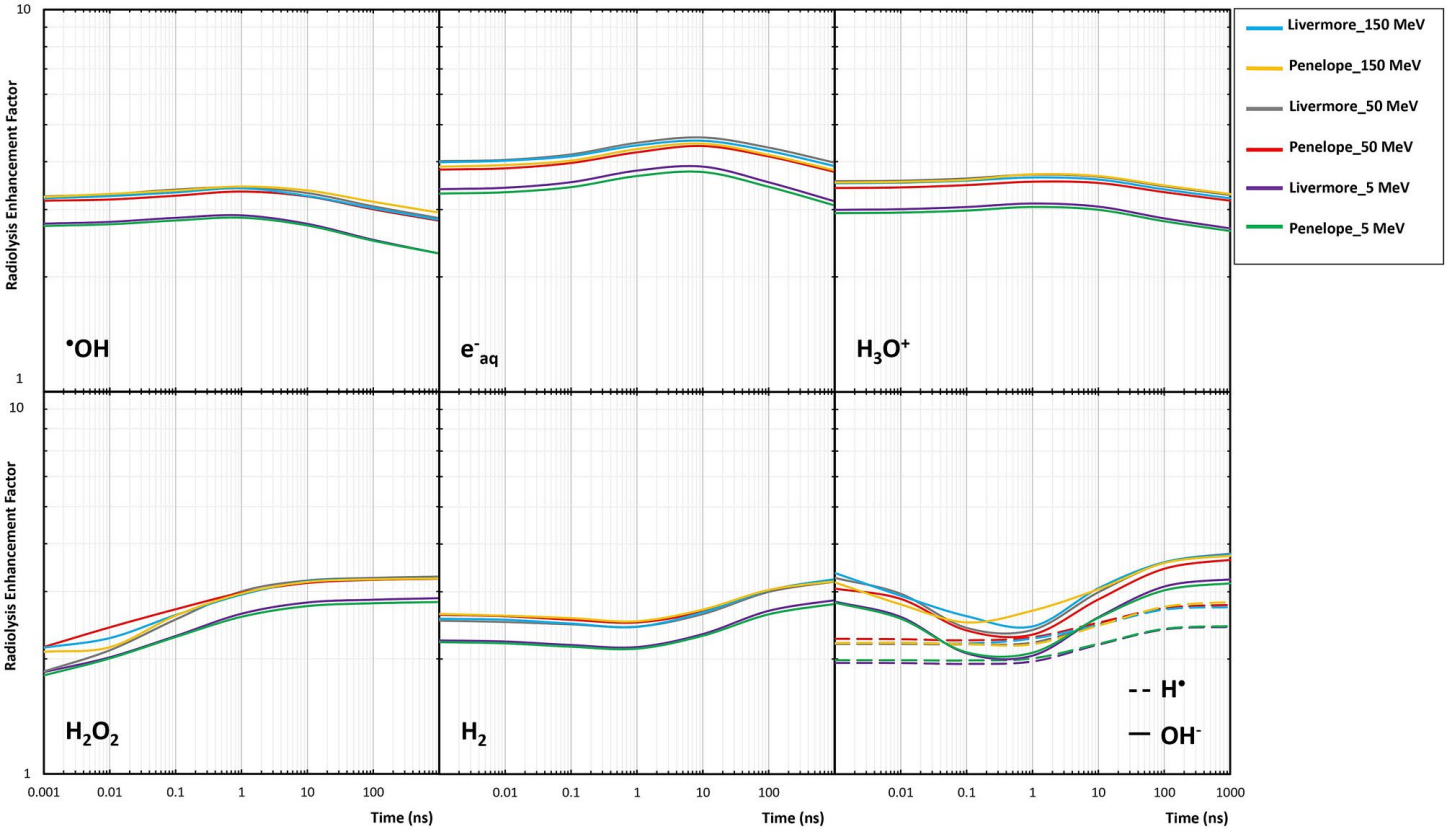
Comparison of the REF between the Livermore and Penelope models obtained for 5, 50, 150-MeV proton beams as a function of time.

Figure 8 illustrates the impact of selecting Livermore and Penelope on the REF of the reactive species of water radiolysis including in the previous section, in the proximity of the GNP irradiated with 5, 50, and 150 MeV incident protons. Also, the differences of G-values and REF obtained by Livermore and Penelope for three proton energies are presented in Table 2. The REF of the two selected models was very similar and independent of physical model selection (within 15%). The REF for all the radiochemical species was always more than 1, which indicated that the G-values induced in the presence of the GNP increased compared with irradiation without the GNP. The REF due to 5 MeV initial proton energy was lower than that of 50 and 150 MeV protons. All the radiochemical species had a slight variation of REF with increasing time, except for OH^−^. The pre-chemical species showed slightly higher REF values than chemical species.

**Table 2:** Differences of the G-value and REF at 1 ps, 1 ns, and 1 µs around GNP between the Livermore and Penelope models obtained for 5, 50, and 150-MeV proton beams.

Species	Time (ns)	5 MeV		50 MeV		150 MeV	
		G-value %Dev.^a^	REF %Dev.^a^	G-value %Dev.^a^	REF %Dev.^a^	G-value %Dev.^a^	REF %Dev.^a^
H_3_O +	0.001	4.368	2.014	5.083	4.217	3.282	0.372
	1	4.463	2.108	5.028	4.329	3.351	1.841
	1000	4.267	1.561	4.767	3.471	3.264	2.594
e_aq_^−^	0.001	4.646	2.693	5.312	5.231	3.563	2.746
	1	4.832	3.342	5.362	5.858	3.952	2.289
	1000	4.617	2.501	5.463	5.724	3.525	2.532
H^·^	0.001	3.182	1.618	3.274	3.061	4.181	0.038
	1	3.548	1.848	3.566	3.121	3.933	3.496
	1000	4.314	0.471	4.789	0.169	3.063	3.049
^·^OH	0.001	4.053	1.342	4.903	2.588	3.504	0.882
	1	4.243	1.407	4.801	2.565	4.052	1.062
	1000	3.725	0.031	4.368	1.515	3.647	4.226
OH^−^	0.001	3.296	0.595	6.554	6.651	1.494	5.854
	1	3.573	1.749	4.311	2.836	1.485	9.062
	1000	4.859	2.437	4.556	3.795	3.987	0.743
H_2_O_2_	0.001	2.828	2.003	0.356	13.73	0.486	2.518
	1	3.838	1.863	4.835	0.843	2.461	0.654
	1000	4.208	2.331	5.181	1.364	2.931	0.159
H_2_	0.001	2.278	1.029	4.647	3.511	3.481	2.814
	1	2.455	0.967	4.436	2.547	3.306	3.306
	1000	3.725	2.301	4.749	0.699	3.823	1.304

^a^Deviation between Livermore and Penelope.

## Discussion

When the proton beam collides with a high atomic number of NPs, the secondary particles including Auger electrons, electrons deriving from ionization process and characteristic X-rays can be emitted from NPs^12^. Furthermore, the subsequent reactive species produced by water radiolysis plays a major role in indirect chemical damages^14,28^. This work aims to quantify the differences between these secondary emissions and radiochemical species by applying various low-energy electromagnetic physics models (Livermore and Penelope) for the GNP region in proton therapy. As presented in Table 1, electrons deriving from ionization process form a large portion of secondary particles at the physical stage of the radiation; the differences between the two models are about 30%. These differences are more obvious at around 200 eV in the secondary electron spectra, as shown in Fig. 3, which can be explained by the significant differences of the Livermore and Penelope models below about 500 eV in electron stopping power and track length within gold material^29^.

The number of Auger electrons through de-excitation processes is very small, thus not contributing significantly to calculating energy deposition based on two selected models. This is in good agreement with the McKinnon et al.’s work, demonstrating less than 1% of dose enhancement accounting for atomic de-excitation. They utilized the Penelope model to simulate the full proton transportation through and outside the ceramic oxide NPs and GNP^30^. Also, Lin et al. showed that activating the auger processes only led to 1.5% dose enhancement^31^. Wälzlein et al. demonstrated that the contribution of X-rays for high Z materials would be less than 0.1%. They also showed that the Auger production leaving from GNPs had energies typically less than 100 eV^32^. This means that Auger electrons emitted from the GNPs were deposited within 10 nm from the gold NP surface^33^. As shown in Table 1, the differences of de-excitation processes indirectly resulted from the different ionization cross-sections for Livermore and Penelope models when Auger electrons and characteristic X-rays occurred following the ionization process^29^. In all the proton energy cases, the X-ray fluorescence yield was larger than the Auger process in GNP, because the probability of emitting fluorescence X-rays strongly increased with atomic number^32^.

Figure 3 shows significant differences of electron spectra based on two selected models at energies between 100 and 250 eV (up to 960%), but up to 30% differences at energies between 250 eV and 1 keV. Although the recommended low energy limit of the Penelope and Livermore physics models are 100 eV and 250 eV, respectively^34^, the Livermore can be used down to 10 eV with the reduced accuracy below ~ 100 eV^35^. The Penelope physics model enables to provide sufficient accuracy at low energies ~ 100 eV^36^; according to the previous study^37^, the uncertainty of the Livermore physics model at energies between 100 eV and 1 keV was about 10–20%.

As mentioned in the result section, the electron spectra had one peak, which originated by the threshold of electron ionization at 100 eV (selected cut-off energy for two models) and proton ionization at 790 eV (mean ionization energy of gold)^13^. This is the limitation of the current Geant4 proton ionization that cannot simulate delta electrons below the mean ionization of materials^13,20^. It should be emphasized that proton tracking models are the same for the Penelope and Livermore models inside the GNP. The number of secondary particles increased with decreasing initial proton energy due to the larger LET of lower proton energies. The influence of model selection on secondary electron spectra was in good agreement with that of the previous study from Sotiropoulos et al.^20^.

The physical model selection only affects the M_α1_ line of characteristic X-rays. These photons have the attenuation length of about 16.2 µm in liquid water^38^ and are expected to influence the distal dose from GNP surface based on two models, which is in good agreement with the study by Tran et al., reporting the contribution of photons after 1 µm from GNP surface^13^. The ionization lines shown in Fig. 4 are the characteristic of the gold material, which were also discussed in the study by Incerti et al.^39^. The differences of bremsstrahlung photon emission up to the factor of 30% are independent of ionization cross-section; however, two selected physical interaction models apply various bremsstrahlung models (the G4LivermoreBremsstrahlungModel and the G4PenelopeBremsstrahlungModel).

The differences in the number of electrons initially produced with two selected models with below 1 keV low energy secondary electrons led to the differences of RDD in the physical models up to a factor of less than 10%. This was in close agreement with the findings of Sotiropoulos et al., which demonstrated the effect of model selection within 20 nm from GNP surface^20^. The behavior of the RDD plots at initial distances from the GNP surface directly depended on the LET of the initial proton beam and, subsequently, on the secondary electron production.

The DEF plots showed an initial increase that is related to the increase of secondary electrons of proton ionization, reaching the value of about 14 which is in good agreement with the previous findings^20,31^. At energies around 100 eV, the DEF values changed rather rapidly due to a larger number of secondary delta electrons with kinetic energies above 790 eV created in the GNP than the water case. Some of the previous simulation studies have compared the Livermore, Penelope, and Geant4-DNA physics models with electron irradiation in pure water. They have revealed that simulations in nanoscale and sub keV energy range strongly depend upon the physics selection, owing to the different inelastic scattering cross-sections of models^34,35^.Differences between the physical models, in terms of DEF, are shown to be negligible (within 15%). According to previous studies differences of about 15% between the models can be considered similar^31,34^. The present findings in the physical phase of irradiation were in good agreement with the recent study from Sotiropoulos et al.^20^. To compare the radiation quantities between the two models and specify their differences, the following expression^35^ was used.

$$ \Delta_{{{\text{rel}}}} \left( \% \right) = \left| {1{\text{-}}Livermore\;value/Penelope\;value} \right| \times 100 $$

Given the indications that production of reactive species by water radiolysis following the physical phase of irradiation plays a significant role in NP-aided proton therapy, it is important to incorporate pre-chemical and chemical phases in simulations and understand the influence of the physical interaction model selection (Livermore and Penelope) on the predicted G-value and REF around GNP. In this simulation study, we focused on the generation of reactive species by secondary electrons emitted from the GNP surface as dominant secondary particles. Tran et al. performed a first simulation study on radiochemical yields around single GNP irradiated with various proton energies and found radiolysis enhancement as a function of distance from GNP surface^13^. Also, Hespeels et al. simulated radial radiolysis enhancement around single GNP with proton irradiation and revealed that larger radiochemical yield is attributable to the larger number of secondary electrons^40^. These studies are consistent with our findings, demonstrating the REF of larger than 1 for all the proton beams and time points. The presence of GNP produced more secondary electron emission than pure water, leading to higher radiolytic yields.

As shown in Fig. 7, the radiolytic yield of hydroxyl radicals, solvated electrons, and hydroniums as products of the pre-chemical phase decreased with increasing time. On the contrary, the yield of hydroxide, dihydrogen, and hydrogen peroxide as a product of the chemical phase after 1 ps directly depended upon the time, which is in good agreement with previous findings^16,41^. The radiolytic yield of all the reactive species decreased with increasing incident proton energy. In contrast, the REF had a direct relation to the incident proton energy with almost similar REF for 50 and 150 MeV energies. These trends originated from the direct relationship of the G-value and REF to the dose and DEF, respectively^13^. The production of reactive species was proportional to secondary electron production and, consequently, the LET of primary proton^40,42^. In this regard, Peukert et al. performed a simulation study to assess the effect of proton energy, NP size, and NP coating features on DEF and REF around single GNP. They revealed that for 5 MeV (high LET) and 50 MeV (lowest LET) protons of those modeled, the REF decreased to a value of less than 8 and 11, respectively^43^. It should be noted that neither the G-value nor the REF was affected by the choice of physical interaction models with the same energy cut-off in the Geant4 (Table 2). Based on the citations that we referenced we assess that a good similarity cutoff for REF is 15%. There are no published simulation data available at the chemical stage which could present the differences of radiochemical yields when Livermore and Penelope interaction models are chosen for GNP.

## Conclusion

In the present study, a simple irradiation scenario of a single GNP with the diameter of 50 nm irradiated in a water phantom by protons with the energies of 5, 50, and 150 MeV was performed by applying the mixed-physics Geant4 simulation toolkit. The physical and chemical quantities around the GNP were compared to investigate the effect of the physical interaction model selection for the NP area. It was found that the RDD, DEF, G-value, and REF were independent of physical model selection within GNP at the same cut-off energies. All of these quantities were directly related to the secondary electron production and, consequently, the incident proton energy. This work illustrated the similarity of the Livermore and Penelope models available in Geant4 for future simulation studies of GNP enhanced proton therapy with physical, physicochemical, and chemical mechanisms. More calculations are needed to verify whether the EM physics models will impact the radiobiological effects such as direct and indirect DNA damages when protons are transported realistically in the GNPs and in the surrounding biological medium.

## Methods

### Simulation geometry

Simulations were performed using Geant4 v10.07.p02. Geant4 was adopted because it is an open-source MC toolkit that enables researchers to create a process for a particle type and perform radiation dosimetry studies in the microscale and nanoscale^19^. The simulations were performed using a computer with a 2.3 GHz 20 core and 20 GB of memory.

**Figure 9:**
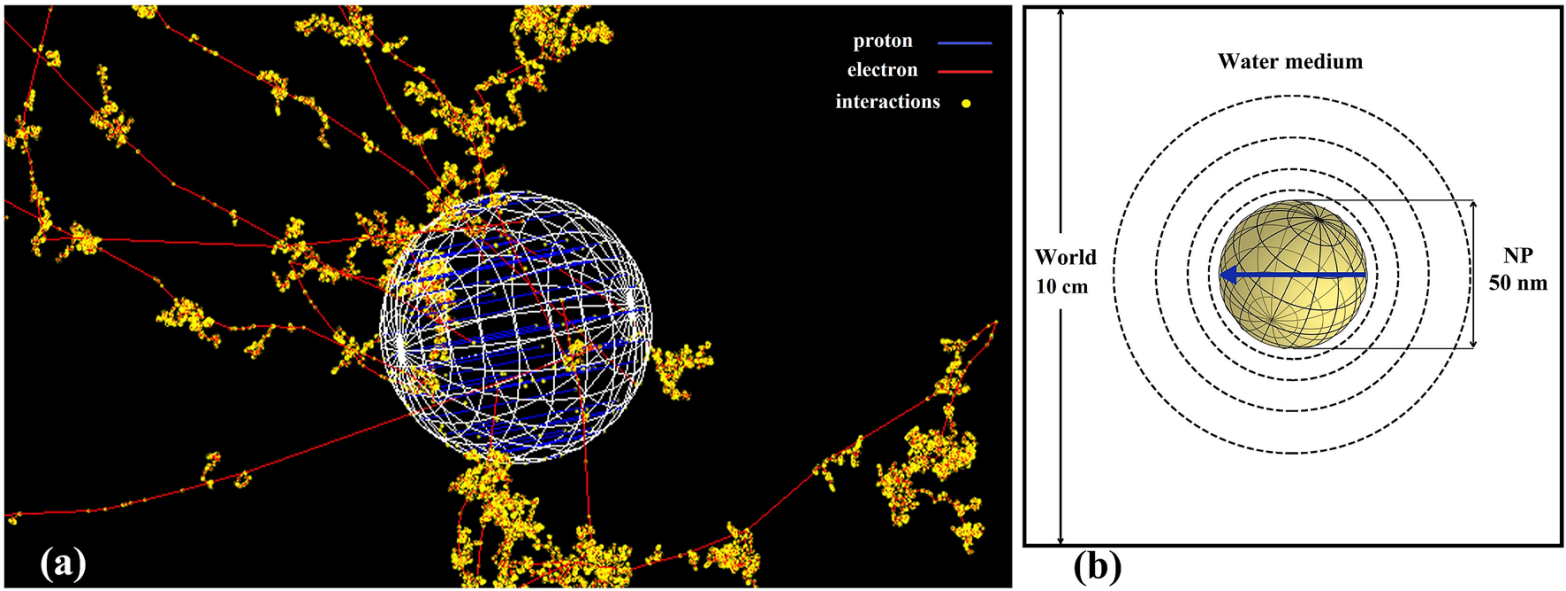
(**a**) Geometry of the Geant4^19^ simulation setup. The parallel proton beams (blue) originating and ending inside the GNP and secondary electron (red) tracks through a 50-nm GNP and the surrounding water medium. (**b**) Schematic diagram of the simulation geometry and the scoring spherical shells (mesh) calculating the RDD and DEF around the GNP.

A single spherical GNP with the diameter of 50 nm was located at the center of the cubic water phantom (10 × 10 × 10 cm^3^) to ensure that no secondary particles and reactive species leave the simulation volume. This diameter was chosen for the GNP because of the high cellular uptake efficiency^44^. The G4GeneralParticleSource (GPS) interface was applied to simulate the proton source. The GNP was irradiated with a monoenergetic proton beam with the energies of 5, 50, and 150 MeV, which were at the energies of therapeutic interest^13^. To quantify the influence of only physical interaction model selection within GNP and reasonable computing time, protons were originated from inside the GNP and killed outside the GNP volume, as shown in Fig. 9a. This irradiation setup was similar to the one adopted in the previous simulation studies^13,20,45^. The RDD was scored in spherical shells with logarithmic thickness from the GNP surface until 1 mm, as shown in Fig. 9b.

### Physical stage

Two low-energy electromagnetic physics models, based on the Penelope and Livermore models, are suitable for the simulation of proton radiation with NPs in Geant4^20^. The Penelope model simulates the tracking of photons, electrons, and positrons in arbitrary materials for the energy ranges of 100 eV to about 1 GeV. The Livermore model allows the simulations of the coupled photon-electron transport down to 10 eV, but the recommended low-energy limit is 250 eV. The Penelope and Livermore models rely upon the condensed history (CH) algorithm. The CH algorithm condenses multiple interactions into a single simulated step, which requires the stopping power of the particles for the energy loss and the multiple scattering theories for the elastic direction changes. The properties of these algorithms include faster simulation with acceptable statistical uncertainty and using various materials^34,46^.

The Geant4-DNA very-low-energy electromagnetic physics processes and model classes, which are valid only in liquid water, allow the tracking of protons, electrons, hydrogen atoms, alpha particles, and their charge states as well as a few ions in the main component of biological medium for the energy ranges of 7.4 eV to about 25 MeV. The Geant4-DNA physics package follows the track structure (TS) algorithms. This formalism explicitly simulates every single interaction in an event-by-event fashion, which requires differential and total cross-sections of the particles with the medium^21,22,34^.

For this study, either the G4EmPenelopePhysics constructor or the G4EmLivermorePhysics constructor named Penelope and Livermore respectively, were applied for the GNP, and the G4EmDNAPhysics_option2 constructor was used for the surrounding water medium. A step size limit of 1 nm and secondary particle production threshold of 100 eV for both models were selected for all the particles^13^. This cut-off energy was chosen for model comparison as an internal threshold in the selected models. The interface of G4VAtomDeexcitation was induced for the simulations of fluorescence, Auger electron production, and particle-induced X-ray emission (PIXE); the production threshold was disabled for the atomic de-excitation. Dose enhancement factor is defined as the ratio of the dose deposited by secondary particles with and without GNP^13^.

$$ {\text{DEF}} = {\text{dose}}\;{\text{deposited}}_{{{\text{with}}\;{\text{GNP}}}} /{\text{dose}}\;{\text{deposited}}_{{{\text{without}}\;{\text{GNP}}}} $$

Additionally, in the case of simulation without GNP, the DEF values were calculated by replacing the GNP with a sphere of the same size, consisting of water with the same physical interaction models as the corresponding GNP. In the physical stage, 10^9^ incident protons were simulated to acquire the acceptable statistical uncertainty of less than 1%.

### Chemical stage

Simulation of the biological effects of proton irradiation requires not only the modeling of the physical stage, but also modeling the chemical stage following physical interactions. The physicochemical and chemical stages in the Geant4-DNA extension were released for the first time in Geant4 version 10.1. These stages simulate the formation, diffusion, and mutual interaction of radiolytic species, as well as their reaction in liquid water based on a step-by-step approach using the Smoluchowski Brownian diffusion equation, which was well-defined in the work by Karamitros et al.^24^.

For this study, the G4EmDNAChemistry_option1 chemistry constructor was selected for the chemistry simulation as well as dynamic time steps based on the probability of the occurrence of radiolytic reactions^16^. Time-dependent yields of the seven important reactive species resulting from water radiolysis including e_aq_^−^, ^·^OH, H^·^, H_3_O^+^, H_2_, OH^−^, and H_2_O_2_ were calculated for an interval from 1 ps up to 1 µs. The G-value represents the number of reactive species generated per 100 eV of deposited energy (molecules/100 eV). Furthermore, the radiolysis enhancement factor was calculated by taking the ratio of radiolysis yield of each reactive species by secondary particles, with and without the GNP^13^.

$$ {\text{REF}} = {\text{G{-}value}}_{{{\text{with}}\;{\text{GNP}}}} /{\text{G{-}value}}_{{{\text{without}}\;{\text{GNP}}}} $$

The combination of this chemistry constructor and the physics constructors was applied to evaluate the influence of physical interactions by comparing G-values and time. In the chemical stage, 10^9^ incident protons were simulated.

### Simulation validation

For the physical stage, the results of the simulated Bragg curve for a proton beam with the energy of 67.5 MeV were compared with the results of the study by Faddegon et al.^27^. For the chemical stage, the results of the simulated time-dependent G-value of hydroxyl radical (^·^OH) were compared with the study of Peukert et al.^14^ for 50-MeV proton beam.
